# Two-colour live-cell nanoscale imaging of intracellular targets

**DOI:** 10.1038/ncomms10778

**Published:** 2016-03-04

**Authors:** Francesca Bottanelli, Emil B. Kromann, Edward S. Allgeyer, Roman S. Erdmann, Stephanie Wood Baguley, George Sirinakis, Alanna Schepartz, David Baddeley, Derek K. Toomre, James E. Rothman, Joerg Bewersdorf

**Affiliations:** 1 Department of Cell Biology, Yale University School of Medicine, New Haven, Connecticut 06520, USA; 2 Department of Biomedical Engineering, Yale University, New Haven, Connecticut 06520, USA; 3 Department of Chemistry, Yale University, New Haven, Connecticut 06520, USA; 4 Gurdon Institute, University of Cambridge, Cambridge CB2 1QN, UK; 5 Department of Molecular, Cellular and Developmental Biology, Yale University, New Haven, Connecticut 06520, USA; 6 Nanobiology Institute, Yale University, West Haven, Connecticut 06516, USA

## Abstract

Stimulated emission depletion (STED) nanoscopy allows observations of subcellular dynamics at the nanoscale. Applications have, however, been severely limited by the lack of a versatile STED-compatible two-colour labelling strategy for intracellular targets in living cells. Here we demonstrate a universal labelling method based on the organic, membrane-permeable dyes SiR and ATTO590 as Halo and SNAP substrates. SiR and ATTO590 constitute the first suitable dye pair for two-colour STED imaging in living cells below 50 nm resolution. We show applications with mitochondria, endoplasmic reticulum, plasma membrane and Golgi-localized proteins, and demonstrate continuous acquisition for up to 3 min at 2-s time resolution.

## Abstract (web summary)

The intracellular applications of STED microscopy are limited by the availability of dyes. Here the authors develop a two-colour labelling strategy based on SiR and ATTO590 dyes, and apply their strategy to image various subcellular membrane compartments.

## Graphical abstract

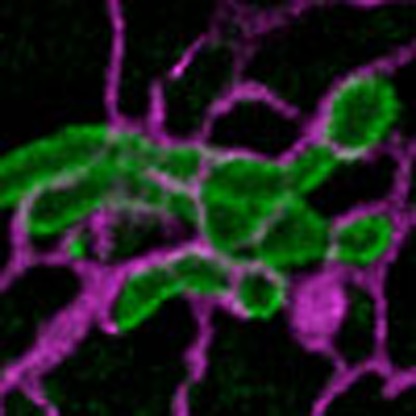

Of the various super-resolution techniques, stimulated emission depletion (STED) nanoscopy^1^ is arguably the most promising for multicolour imaging of rapid nanoscale dynamics in living cells^2^. Previous demonstrations of multicolour live-cell STED nanoscopy failed, however, to translate into widespread application due to limitations of the available fluorophores. This is particularly true for the investigation of intracellular dynamics because of a lack of STED-compatible membrane-permeable dyes. To date, the only feasible option for two-colour intracellular labelling of living cells for STED nanoscopy has been the green fluorescent protein/yellow fluorescent protein combination, which shows significant crosstalk, requires post-acquisition image processing and is less photostable than most organic fluorescent dyes^3^,^4^. Recently, brighter and more photostable dyes have pushed multicolour STED image quality to a new level in fixed cells, enabling resolutions as good as 20 nm in multiple colour channels^5^. However, most STED-compatible dyes are not membrane permeable and cannot access intracellular structures in intact living cells. For this reason these dyes have primarily been used to visualize endocytic events at the plasma membrane^6^ or to localize extracellular proteins in neurons^7^,^8^,^9^.

The recent introduction of the membrane-permeable dye silicon–rhodamine (SiR) represents a major step forward for live-cell STED imaging^7^,^10^,^11^,^12^, but a membrane-permeable dye suitable for use as a second colour has remained elusive. The only other known STED-compatible and membrane-permeable dye, tetramethylrhodamine (TMR), has also been used in single-colour STED experiments^13^. Unfortunately, TMR and SiR require depletion at different wavelengths^10^,^13^, which makes this dye pair incompatible for two-colour live-cell STED nanoscopy where both colour channels have to be recorded repeatedly. The necessity of finding a STED-compatible dye, which is spectrally distinct from SiR but depletes at the same wavelength, led us to screen a range of dyes with excitation peaks around 590 nm (ref. 5). To do so, we took advantage of Halo and SNAP tags^14^,^15^, which rapidly and specifically react with chloroalkane (CA) and benzylguanine (BG) derivatives, respectively, to covalently label intracellular proteins of interest. We discovered that Halo and SNAP substrates of the rhodamine dye ATTO590 can cross the membrane of living cells, providing the critical second colour needed to complement SiR for two-colour live-cell experiments. Here we present the results of testing this dye combination with markers of various intracellular organelles and show validation of the general applicability of this labelling strategy in STED nanoscopy of living cells.

## Results

### Dynamics of mitochondria and the endoplasmic reticulum

**Figure 1:**
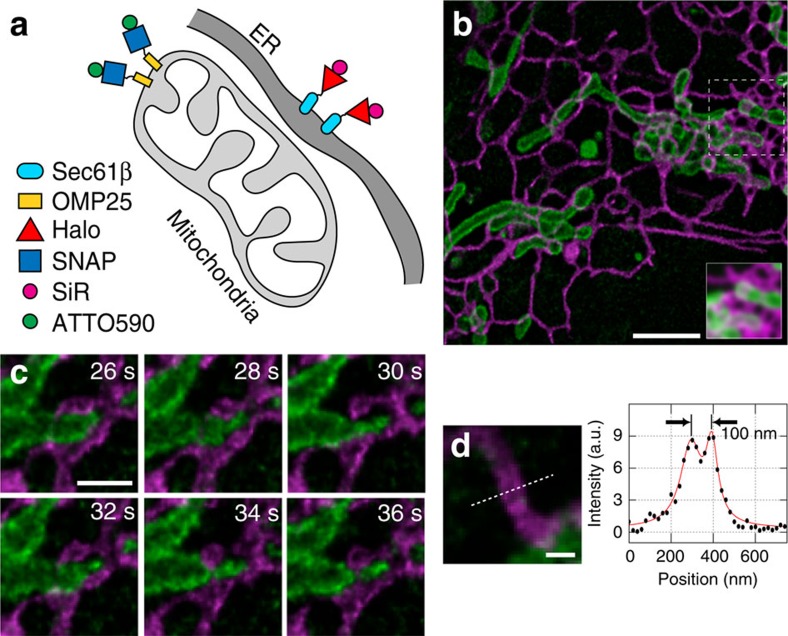
STED nanoscopy of dynamic interactions between ER and mitochondria. (**a**) Strategy for labelling ER (Halo-Sec61β with SiR-CA, magenta) and mitochondria (SNAP-OMP25 with 590-BG, green). (**b**) The first frame from a live-cell STED image sequence of a correspondingly labelled COS-7 cell. For comparison between STED and confocal imaging, the inset shows a Gaussian-blurred (full-width at half-maximum=250 nm) version of the raw image data in the boxed region. (**c**) Region of interest showing a dynamic interaction between the ER and a mitochondrion. (**d**) A line profile through an ER tubule demonstrates that the lumen of the ∼100-nm-wide tubule can be distinguished from its membrane. Scale bars, **b**=2 μm; **c**=500 nm; **d**=200 nm. Images were derived from the same image sequence. Imaging was performed at 37 °C. All images were corrected for bleaching and deconvolved, while the line profile represents raw image data.

Using Halo-reactive SiR-chloroalkane (SiR-CA) and SNAP-reactive ATTO590-benzylguanine (590-BG), we labelled living cells expressing Halo-Sec61β and SNAP-OMP25 decorating the endoplasmic reticulum (ER) and the outer mitochondrial membrane, respectively (Fig. 1a,b and Supplementary Movie 1). Cells were imaged on a custom-built STED nanoscope with 594 and 650 nm excitation lasers (Supplementary Fig. 1) capable of 20–30 nm resolution in fixed samples (Supplementary Fig. 2) and sub-50 nm in living specimens (Supplementary Fig. 3). Fixed-cell imaging demonstrates negligible crosstalk between the two detection channels (Supplementary Fig. 4). In living cells, this dye combination allows the acquisition of one two-colour image every 2 s, with good image quality. This enabled us to observe dynamic events, such as ER tubules making contact with mitochondria^16^, with unprecedented detail. In Fig. 1c, we highlight an ER tubule possibly constricting and shaping a mitochondrion (Supplementary Movie 2). In addition, the two sides of a hollow ER tubule are distinguishable (Fig. 1d). In contrast to the unspecific mitochondrial staining of ATTO647N in living cells^6^, no unspecific binding to intracellular membranes and only very little background due to endocytosis of the dyes was observed (Supplementary Fig. 5).

### Dynamics of Golgi-localized Halo- and SNAP-fusion proteins

**Figure 2:**
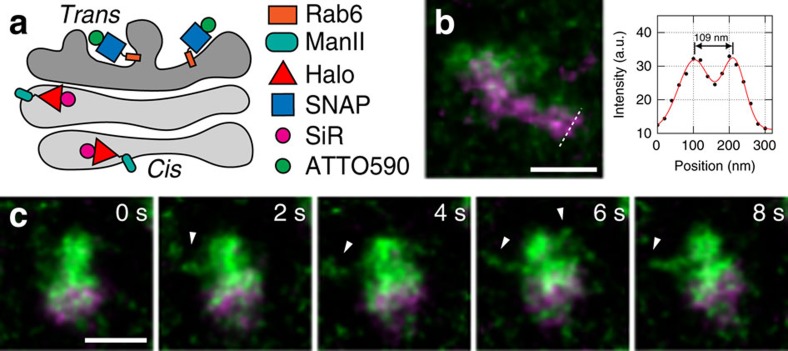
Golgi protein dynamics imaged by live-cell STED nanoscopy. (**a**) Strategy for labelling *cis/medial*-Golgi (ManII-Halo with SiR-CA, magenta) and *trans*-Golgi (SNAP-Rab6 with 590-BG, green). (**b**) A line profile through a correspondingly labelled Golgi ministack in a nocodazole-treated HeLa cell shows two distinct structures within the *cis/medial*-Golgi (potentially, separate cisternae). (**c**) Image sequence showing transient tubular structures on a Golgi ministack as indicated by white arrowheads. Scale bars, 500 nm. Imaging was performed at 37 °C. All images were corrected for bleaching and deconvolved, while the line profile represents raw image data.

The general applicability of our labelling strategy is emphasized by the observation of protein dynamics at the Golgi (Fig. 2). Cells were treated with the microtubule-depolymerizing drug nocodazole to break down the Golgi ribbon and facilitate the visualization of ministacks in the periphery of the cell^17^. We observe a clear separation of the *cis/medial*-Golgi cisternae labelled with SiR-CA on Mannosidase II-Halo (ManII-Halo)^18^ and the *trans*-Golgi cisternae labelled with 590-BG on SNAP-Rab6 (Fig. 2 and Supplementary Fig. 6). Moreover, we can discern two separate structures, possibly individual Golgi cisternae, only ∼110 nm apart (Fig. 2b and Supplementary Movie 3). We also observe transient tubular structures labelled by SNAP-Rab6 (Golgi-to-plasma membrane carriers^19^) protruding from *trans*-Golgi cisternae (Fig. 2c and Supplementary Movie 4).

### Dynamics of clathrin and transferrin receptor

**Figure 3:**
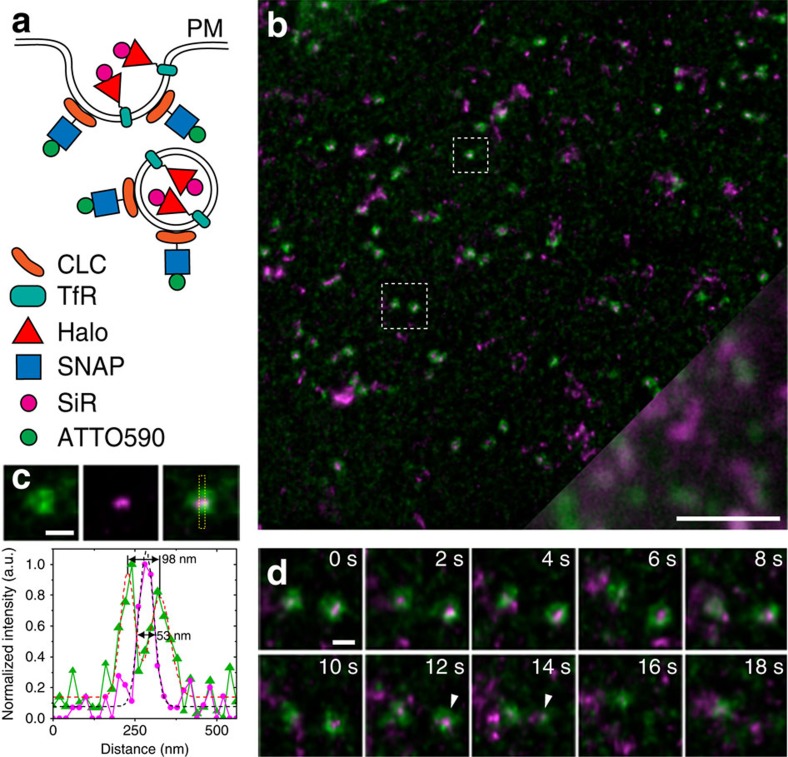
STED nanoscopy imaging of CCPs at the plasma membrane (PM). (**a**) Strategy for labelling CCPs (SNAP-CLC with 590-BG, green) and the endocytic cargo transferrin receptor (TfR-FM4-Halo with SiR-CA, magenta). (**b**) First frame from a live STED image sequence of COS-7 cells. The bottom right corner shows the comparison between STED and an equivalent field of view imaged in confocal mode. (**c**) Magnified region of interest (small box in **b**) with line profile as indicated by yellow box. (**d**) Region of interest (large box in **b**) showing a putative clathrin-mediated endocytic event (indicated by arrowheads). Scale bars, **b**=2 μm; **c**,**d**=200 nm. Imaging was performed at 22 °C. All images were corrected for bleaching and deconvolved, while the line profile represents raw image data.

To further demonstrate the capabilities of the ATTO590/SiR dye pair, we imaged endocytic events at the plasma membrane. SNAP-tagged clathrin light chain (SNAP-CLC) and Halo-tagged transferrin receptor (TfR-FM4-Halo) were, respectively, labelled with 590-BG and SiR-CA (Fig. 3a). Our STED movies clearly resolve the hollow centres of clathrin-coated pits (CCPs) as demonstrated by the example in Fig. 3c, where we show a ring-like structure of ∼100 nm diameter surrounding the endocytic cargo TfR-FM4-Halo (Fig. 3b,c and Supplementary Movie 5). Figure 3d shows a putative endocytic event. While the left CCP persists almost until the end of the image sequence, the right CCP disappears, suggesting internalization.

### Labelling membrane and lumen of the endoplasmic reticulum

**Figure 4:**
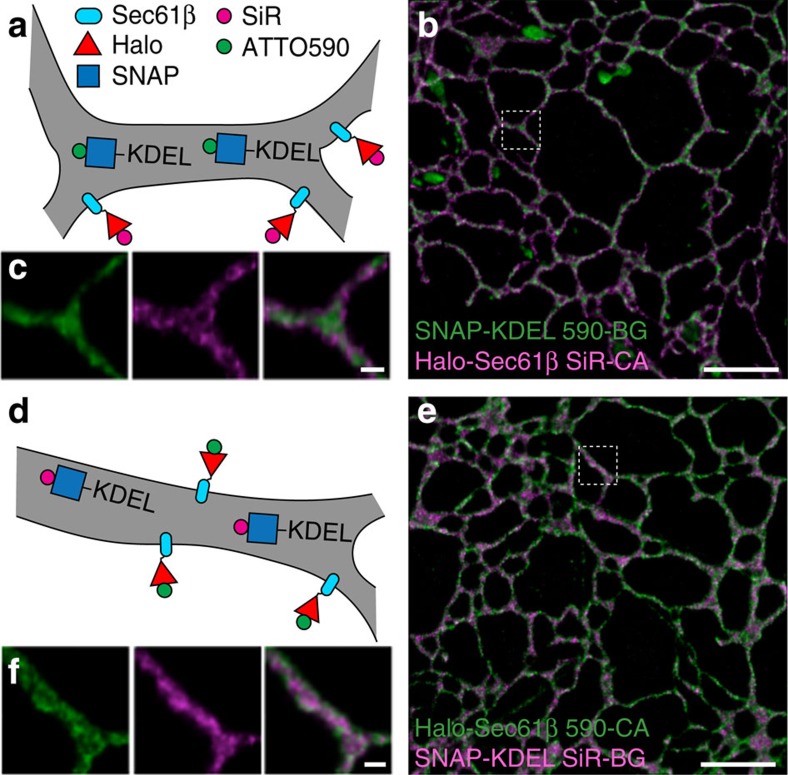
Two-colour STED nanoscopy of the ER. (**a**,**d**) Strategy for labelling ER membrane (Halo-Sec61β) and ER lumen (SNAP-KDEL). COS-7 cells expressing the above mentioned fusion proteins were then labelled with 590-BG and SiR-CA (**a**–**c**) and 590-CA and SiR-BG (**d**–**f**). (**c**,**f**) Magnified regions of interest (indicated by white boxes) from **b** and **e**, respectively. Scale bars, **b**,**e**=2 μm; **c**,**f**=200 nm. Imaging was performed at 22 °C. All images were deconvolved and corrected for bleaching.

Finally, to further demonstrate the resolution capabilities of two-colour live-cell STED nanoscopy, we labelled the ER membrane with Halo-Sec61β and the ER lumen with SNAP carrying the C-terminal tetrapeptide KDEL (SNAP-KDEL), which retains proteins in the ER lumen^20^. We then labelled these fusion proteins with SiR-CA and 590-BG (Fig. 4a–c and Supplementary Movie 6) as well as the reverse labelling scheme 590-CA and SiR-BG (Fig. 4d–f and Supplementary Movie 7). In both cases, we can distinguish the lumen from the membrane of the ER (Fig. 4c,f). Particularly, for the 590-Halo-Sec61β and SiR-SNAP-KDEL combination, it was possible to image 90 sequential two-colour frames with a two-colour frame rate of 0.5 s^−1^, thus demonstrating the feasibility of acquiring long (∼3 min) image sequences with two-colour live-cell STED nanoscopy.

## Discussion

Until now, multicolour live-cell imaging was primarily the domain of diffraction-limited light microscopy. We have introduced a novel combination of cell-permeable dyes, which enables the general application of two-colour STED nanoscopy, to study intracellular dynamics.

Our method expands on previous pioneering experiments of two-colour STED nanoscopy in living cells. Tønnesen *et al*.^3^ used fluorescent proteins (green fluorescent protein and yellow fluorescent protein) as well as injected organic dye pairs (Alexa Fluor 488 and calcein green) as markers of different neuronal populations. Samples were imaged with a spatial resolution of about 80 nm, and one frame was acquired every 10 min. Using linear unmixing between two detection channels, Tønnesen *et al*. showed that a green-emitting fluorophore pair can be imaged with a single excitation laser in STED nanoscopy (thus, lowering sample exposure). Linear unmixing requires, however, a very good signal-to-noise ratio, making it challenging to image two dim structures in close proximity^3^,^4^. In a previous study from our group, a long Stokes shift dye (Chromeo494) was used together with ATTO647N to label extracellular structures on the surface of living cells^6^, enabling acquisition of 22 consecutive two-colour images at about 80 nm resolution, each image recorded in 11.9 s. This approach was, however, hampered by the lack of membrane permeability of the used dyes. More recently, D'Este *et al*.^7^ imaged the subcortical cytoskeleton in living neurons, using the commercially available SiR-actin probe and antibodies carrying the dye STAR580 to label extracellular fragments of neuronal proteins. D'Este *et al*. demonstrate excellent image quality but report only single frames.

Our new approach has major advantages over previous work. First, our labelling approach and dyes all work well with intracellular targets. Both dyes are membrane-permeable and produce little to no unspecific background staining in our experiments. Using SNAP and Halo tags warrants general applicability as demonstrated by the wide range of examples shown. Second, the used fluorophores are among the best STED dyes currently known. They are photostable, especially when compared with fluorescent proteins (Supplementary Fig. 7) and provide excellent STED resolution. In this work, we have demonstrated continuous (up to 90 frames) imaging of two intracellular targets with a spatial resolution below 50 nm (Supplementary Fig. 3). From imaging fluorescent beads, we know that our custom-built STED nanoscope can attain a resolution in the 20–30 nm range (Supplementary Fig. 2). In our live-cell images, resolution was limited to 40 nm as dictated by the Nyquist sampling criterion and the 20 nm pixel size used. This pixel size was chosen as a tradeoff between pixel size, field of view, signal-to-noise ratio and frame rate (Supplementary Note 1). If a higher bleaching rate is acceptable, higher resolution should be attainable with the same probes.

Excitation and depletion lasers in the red wavelength range, as used by D'Este *et al*. currently provide the best STED image quality, as nicely demonstrated by Göttfert *et al*.^5^ While the laser configuration in our STED nanoscope is inspired by the same work, we additionally implemented a fast-scanning resonance mirror (16 kHz), markedly reducing the pixel dwell time relative to conventional sample or beam scanning. Shorter pixel dwell times enable faster imaging, can increase the photon yield and reduce photobleaching^21^,^22^. This feature contributed to the demonstrated acquisition of relatively long image sequences at a high frame rate.

The focused and relatively high-powered STED laser (140 mW in the objective pupil) raises concerns whether and how phototoxicity may disrupt the natural behaviour of the cells being imaged. For the image sequences presented here (<5 min), long-term adaptations are not an immediate concern and we see no indication of an acute phototoxic response. As fast scanning reduces photobleaching, it likely also reduces other types of photodamage^21^. Moreover, the long pulse length (∼600 ps) of our STED laser results in a much lower peak power than the peak powers used in, for example, two-photon microscopy. In addition, the red-shifted laser configuration makes absorption by endogenous molecules less likely, and the lower photon energy should decrease the risk of photodamage from single-photon interactions^23^. Future quantitative studies of photodamage, in particular for longer-term STED imaging (>5 min), will be very valuable to identify the limits of live-cell STED imaging in this regard.

In summary, we have demonstrated that the dye pair ATTO590 and SiR extends intracellular live-cell STED imaging to multicolour imaging. This development is of high importance to the community since the used dyes are fully compatible with current commercial STED nanoscopes. The presented advancement in multicolour live-cell nanoscopy with sub-50 nm resolution enables researchers to address a large range of questions in cell biology that concern subcellular interactions at the tens of nanometre scale in dynamic systems.

## Methods

### Cell culture and labelling of live cells

Monkey fibroblast-like kidney cells (COS-7 ATCC CRL-1651) and HeLa cells (ATCC CCL-2) were grown at 37 °C in 5% CO_2_ in DMEM (Gibco) supplemented with 10% FBS (Gibco). Cells were seeded on a glass-bottom dish (MatTek, 3.5 cm diameter, No. 1.5), previously cleaned by sonication (1 M KOH for 15 min) and coated with fibronectin (Chemicon), and transfected using Lipofectamine 2000 (Invitrogen) following the manufacturer's instructions. Details of recombinant plasmids are provided in Supplementary Note 2. Cells were labelled 14–16 h after transfection, with 5 μM of the various SNAP (BG) and Halo (CA) substrates^24^ (SNAP-Cell 647-SiR (New England BioLabs), SiR-CA (a gift from Promega), 590-BG and 590-CA (Supplementary Notes 2 and 3)) for 1 h. After extensive washes, cells were left to wash out the excess dye for 2–3 h. For the labelling of the Golgi, cells were treated with verapamil (a broad-spectrum efflux-pump inhibitor) during the labelling reaction to enhance the staining^11^,^25^, and then with 2 μg ml^−1^ nocodazole (Sigma) during the washout time. TfR-FM4-Halo is initially aggregated in the lumen of the ER, and the release to the plasma membrane and endosomes is triggered by addition of D/D Solubilizer (Clontech) for 1 h before imaging.

### Custom-built STED nanoscope

As shown in Supplementary Fig. 1, an 80-MHz pulsed depletion laser with a pulse length of ∼600 ps (775 nm, Katana, OneFive) is coupled into a 100-m long polarization maintaining single-mode fibre (PM-SMF) (PM630-HP, Thorlabs). The beam emerging from the fibre is collimated and illuminates a spatial light modulator (X10468-02, Hamamatsu), which applies a helical phase ramp to the depletion beam and enables aberration correction within the depletion laser beam path. The spatial light modulator is imaged onto a 16-kHz resonance mirror (SC-30, EOPC), which, in turn, is imaged into the pupil of a 100 × oil immersion objective lens (UPLSAPO 100XO PSF, Olympus), mounted in an inverted microscope stand (IX71, Olympus). Three pulsed excitation lasers (485 nm, LDH-P-C-485B, PicoQuant; 650 nm, LDH-D-C-650, PicoQuant; 594 nm, LightUp594, Abberior or alternatively LDH-D-TA-595, PicoQuant) are coupled into a single 2-m long polarization maintaining single-mode fibre (P1-488PM-FC-2, Thorlabs) and merged with the STED beam path via a dichroic mirror (zt750spxr, Chroma). The resonance mirror scans the combined laser beams along the fast scanning axis, while two synchronized galvanometer scanning mirrors (dynAXIS XS, SCANLAB) scan the beams along the slow scanning axis. Together, the two galvanometer mirrors imitate the scanning of a single mirror at the conjugated pupil plane, allowing the three scanning mirrors to act as a fast, dual-axis scanning system positioned in a plane conjugate to the pupil plane of the objective. Circular polarization of all lasers is ensured by a half-wave plate in the depletion laser beam path (RAC 5.2.10, B. Halle Nachfl. GmbH), a half-wave plate (AHWP05M-600, Thorlabs) in the excitation laser beam path and a quarter-wave plate (AQWP05M, Thorlabs) in the common beam path. Fluorescence emitted from the sample is collected by the objective, de-scanned by the three scanning mirrors and then separated from the excitation laser beam paths via a custom-made 5-mm-thick quad-bandpass dichroic mirror (zt485/595/640/775rpc, Chroma). Additional dichroic mirrors (zt568rdc, zt640rdc, Chroma) split the fluorescence into three detection bands. For each detection band, two filters of the same type reject stray excitation and depletion laser light (FF03-525/50–25; FF01-624/40–25; FF02-685/40–25, Semrock). Fluorescence in each detection band is coupled into a multimode fibre (AFS105/125Y, Thorlabs) acting as a confocal pinhole, with pinhole sizes of 0.7–0.8 Airy units. Each multimode fibre is connected to an avalanche photodiode (SPCM-ACRH-13-FC, Excelitas). The measured fluorescence signals from the avalanche photodiodes are relayed to custom-made circuit boards for gated detection (Opsero Electronics). These gate boards are synchronized to the trigger signal from the depletion laser, also used to trigger the excitation lasers. The gate electronics allow software control of the detection window length and position. Finally, the fluorescence signal is acquired by a field-programmable gate array board (PCIe-7852R, National Instruments), which is synchronized to the resonance mirror. The system is controlled via a custom-made interface programmed in LabVIEW (National Instruments). The system allows line-by-line image acquisition. One line of the first colour channel is followed by the acquisition of one line for the second colour channel, thus enabling quasi-simultaneous two-colour imaging.

### Imaging parameters

While the set-up allows to tune the size of the imaged region, all live-cell images shown in the main text were recorded using line-by-line image acquisition (described above) and a 10.24 × 10.24-μm field of view (512 × 512 pixels and 20 nm pixels). These acquisition settings result in an average pixel dwell time of ∼40 ns (per scan of the resonance mirror) due to the 16-kHz scan rate. We used unidirectional acquisition, meaning that we only recorded the fluorescence signal during the forward scan of the resonance mirror. Each line in the shown images was scanned 32 times per frame and per colour channel, resulting in a total pixel dwell time of ∼1.28 μs for each colour channel and an acquisition time of ∼2 s for each two-colour frame. Frames were acquired continuously, with a negligible wait time between frames (<10 ms) as the galvanometer mirrors returned the laser focus from the bottom to the top of the field of view. All live-cell images (Figs 1, 2, 3, 4 and Supplementary Movies 1–7) were recorded with excitation laser powers of ∼20 μW and a STED laser power of ∼140 mW in the objective pupil. To measure these laser powers, we placed an iris with an open diameter of 5.04 mm (corresponding to the pupil diameter of the objective) in the objective turret and then measured the laser powers at this position. Measurements were made with the lasers running continuously (beam blanking for the non-imaging return scan of the resonance mirror was deactivated) using a photodiode power sensor (S130A, Thorlabs) for the excitation lasers and a thermal power sensor (S310C, Thorlabs) for the STED laser.

For completeness, we report the following differences between the image acquisition settings for different figures in the main text: data for Figs 1 and 2 were recorded at 37 °C using a stage-top incubator (DH-35iL, Warner). We used an excitation laser emitting at 594 nm (PDL-594, Abberior Instruments) and did not blank the lasers during the non-imaging return scan of the resonance mirror. Data for Figs 3 and 4 were recorded at 22 °C (room temperature); we used an excitation laser emitting at 595 nm (LDH-D-TA-595, PicoQuant) and blanked all lasers during the non-imaging return scan of the resonance mirror, which reduces bleaching of the sample by about a factor of two.

All STED images were slightly deconvolved using a Richardson–Lucy algorithm primarily to reduce noise (see Supplementary Note 2 for details).

## Additional information

**How to cite this article:** Bottanelli, F. *et al*. Two-colour live-cell nanoscale imaging of intracellular targets. *Nat. Commun.* 7:10778 doi: 10.1038/ncomms10778 (2016).

## Supplementary Material

Supplementary Information Supplementary Figures 1-7, Supplementary Notes 1-3 and Supplementary References

Supplementary Movie 1 Live-cell imaging of mitochondria and ER in COS7 cells shown in Figure 1b. Scale bar = 2 µm. This movie was corrected for bleaching and deconvolved.

Supplementary Movie 2 Live-cell imaging of mitochondria and ER in COS7 cells shown in Figure 1c (crop from Fig. 1b). Scale bar = 500 nm. This movie was corrected for bleaching and deconvolved.

Supplementary Movie 3 Live-cell imaging of a Golgi ministack in nocodazole-treated HeLa cells shown in Figure 2b. Scale bar = 500 nm. This movie was corrected for bleaching and deconvolved.

Supplementary Movie 4 Live-cell imaging of a Golgi ministack in nocodazole-treated HeLa cells shown in Figure 2c. Scale bar = 500 nm. This movie was corrected for bleaching and deconvolved.

Supplementary Movie 5 Live-cell imaging of CCPs in COS7 cells shown in Figure 3b. Scale bar = 2 µm. This movie was corrected for bleaching and deconvolved.

Supplementary Movie 6 Live-cell imaging of ER in COS7 cells shown in Figure 4b. Scale bar = 2 µm. This movie was corrected for bleaching and deconvolved.

Supplementary Movie 7 Live-cell imaging of ER in COS7 cells shown in Figure 4e. Scale bar = 2 µm. This movie was corrected for bleaching and deconvolved.
