# Supplementary material for: Two-colour live-cell nanoscale imaging of intracellular targets
Source: Nat Commun. 2016 Mar 4;7:10778. doi: 10.1038/ncomms10778 (PMC4785223; doi:10.1038/ncomms10778)
Supplement: Supplementary Information — Supplementary Figures 1-7, Supplementary Notes 1-3 and Supplementary References [file ncomms10778-s1.pdf]

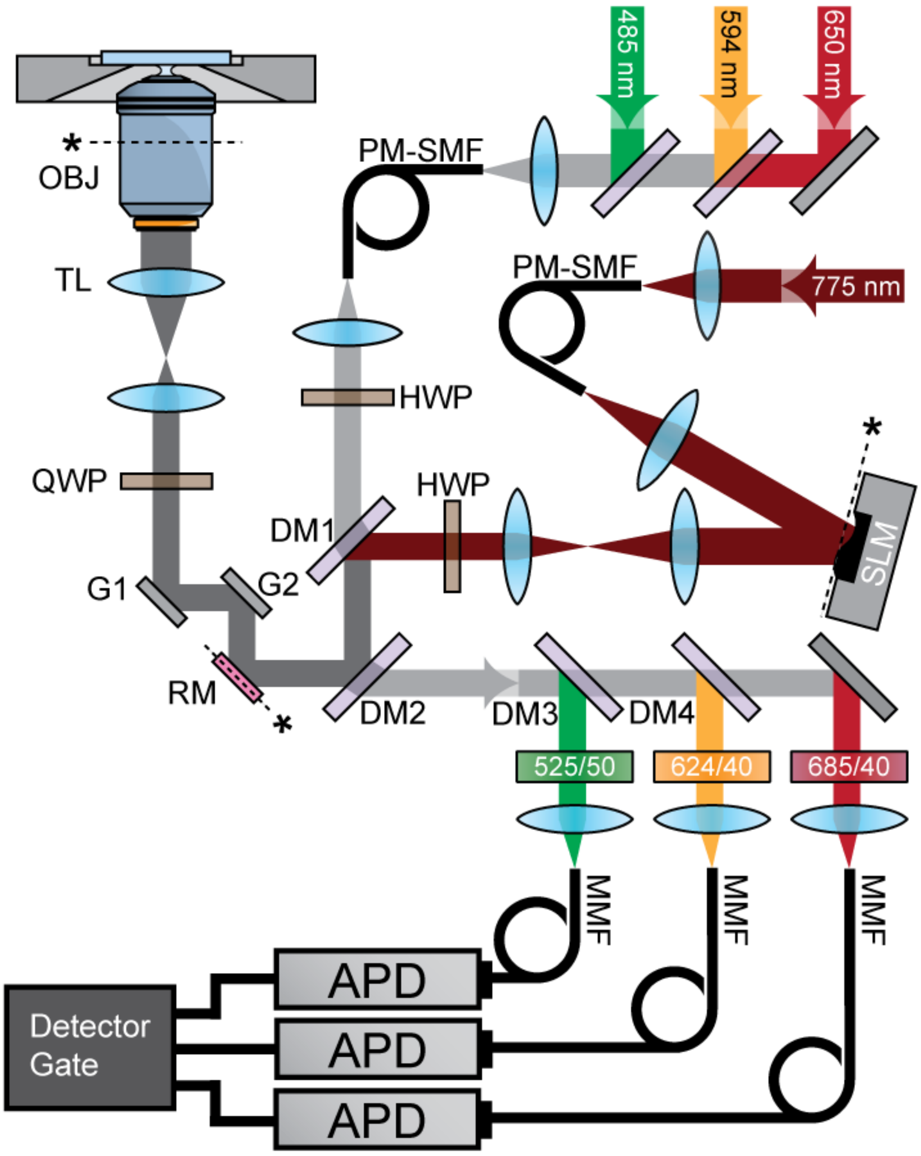

Supplementary Figure 1: Simplified schematic of custom-built STED nanoscope. The system is built from the following components: Excitation lasers (485 nm, 594 nm, 650 nm), depletion laser (775 nm), polarization-maintaining single-mode fiber (PM-SMF), multi-mode fiber (MMF), dichroic mirror (DM), half-wave plate (HWP), quarter-wave plate (QWP), spatial light modulator (SLM), resonance mirror (RM), galvanometer scanning mirror (G), tube lens (TL), objective (OBJ), detection filters (525/50, 624/40, 685/40), avalanche photo diode (APD), and detector gate electronics. Asterisks (*) indicate planes conjugate to the pupil of the objective.

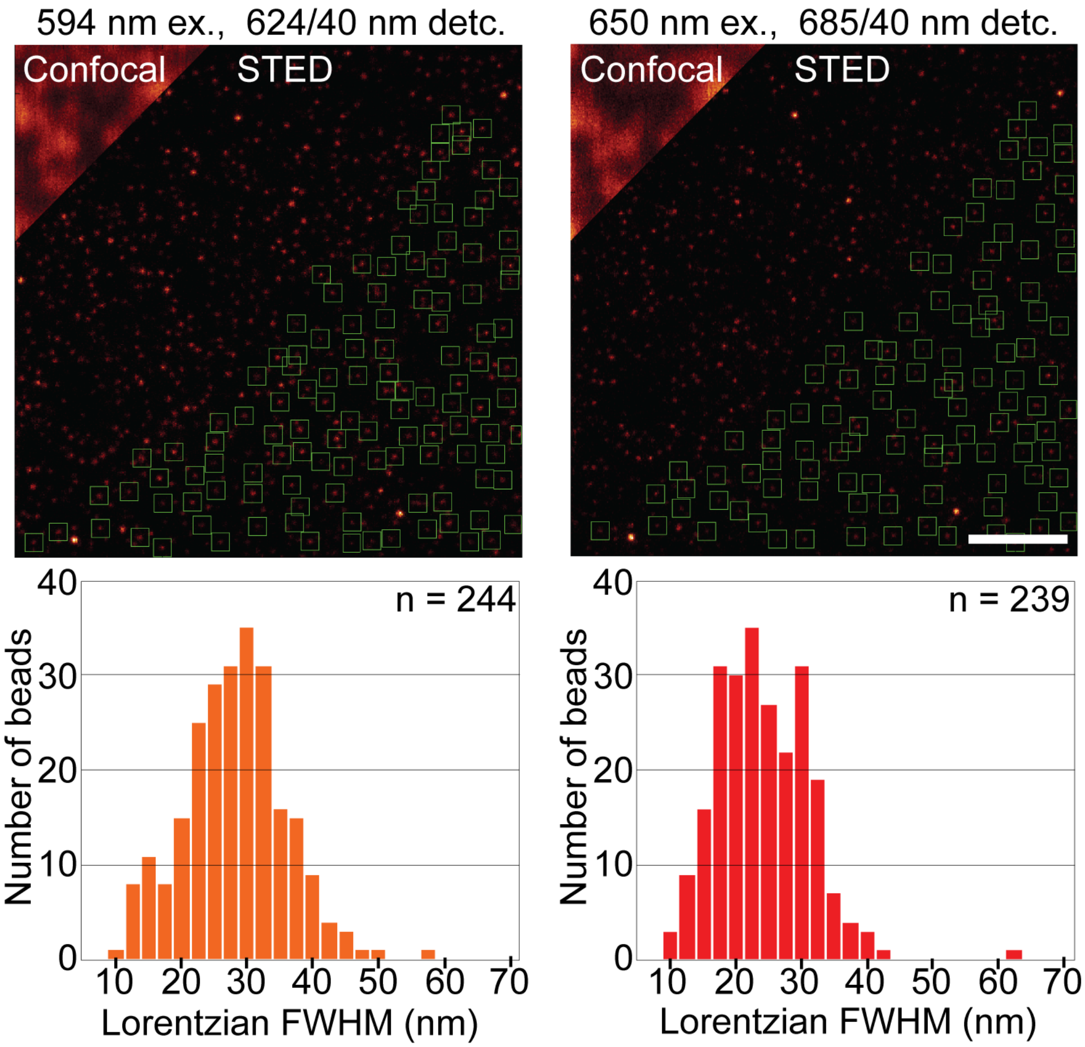

Supplementary Figure 2: Quantification of image resolution. Crimson fluorescent beads with 20 nm diameter (Invitrogen) were imaged in conventional confocal and STED mode. Left: Imaging configuration used to image ATTO590. Right: Imaging configuration used to image SiR. A two-dimensional Lorentzian function was fitted to images of isolated beads (green boxes, only shown in one corner of each image). The full width at half maximum (FWHM) of the fitted functions are represented in histograms. *n* is the number of fitted beads. Bin-width = 2.5 nm. Scale bar = 1 µm. Based on these histograms, we estimate a resolution in the range of 20-30 nm for both imaging configurations. Laser powers and exposure time were optimized for the near-ideal bead-sample with crimson dye. Excitation laser powers were 5-15 µW and the STED laser power was ~250 mW measured in the objective pupil. Each line in the images (512 x 512 pixels of 10 nm size) was scanned 600 times, resulting in a ~40 ns pixel dwell time per scan of the resonance mirror and a total pixel dwell time of ~24 µs. Each single-color image was acquired in ~19.2 s.

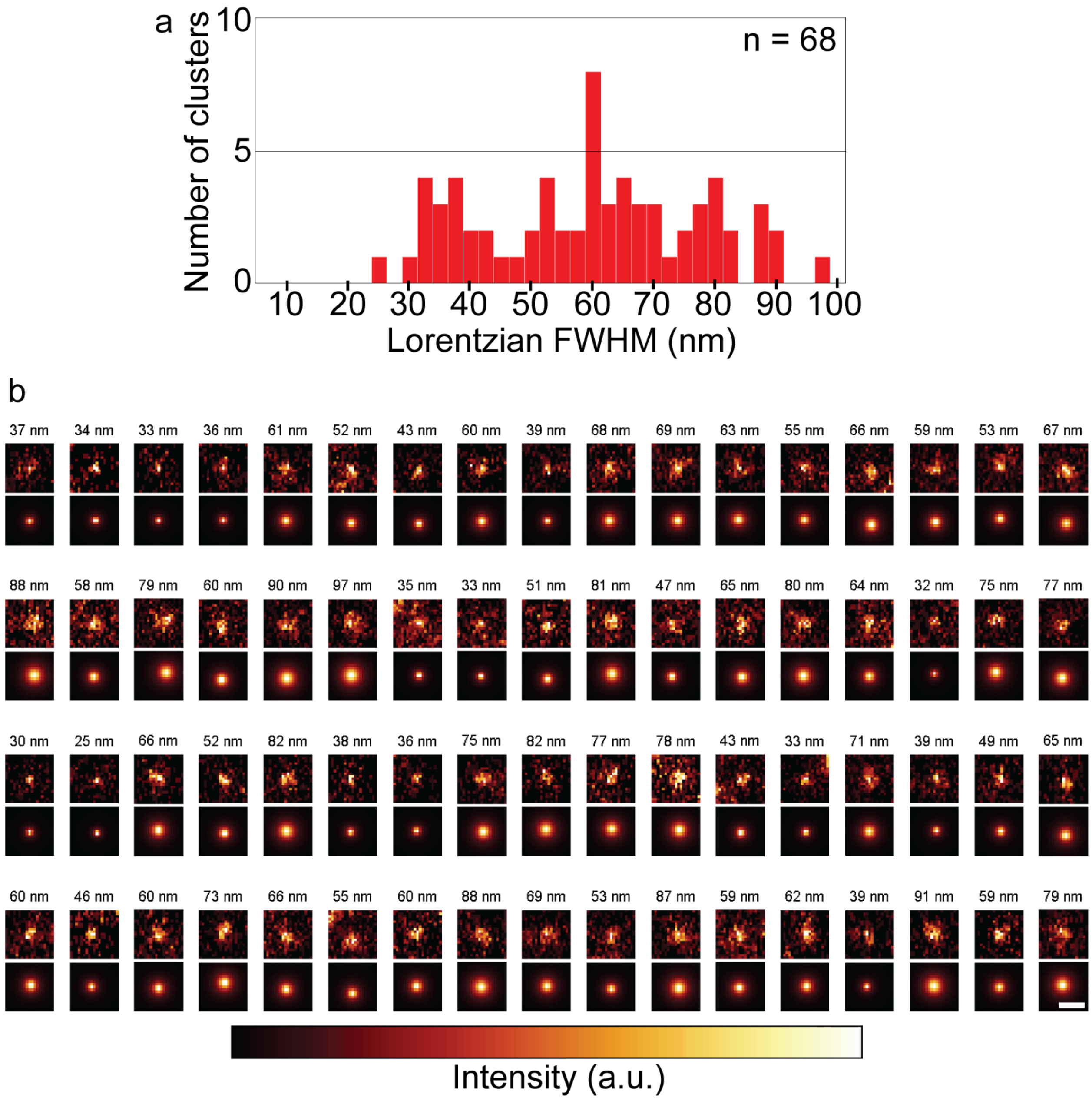

Supplementary Figure 3: Quantification of resolution in live-cell experiments. Estimating the resolution directly from live-cell STED data is challenging due to the absence of easily identifiable sub-resolution sized structures. Here, we estimate the image resolution in our live-cell movies, from images of SiR-labeled transferrin receptors (TfR-FM4-Halo with SiR-CA), which form dot-like clusters during endocytosis. We used the same imaging settings and labeling strategy as in **Figure 3** and **Supplementary Movie 5**. We manually selected 68 clusters in five different cells. As in **Supplementary Figure 2**, each cluster was fitted with a two-dimensional Lorentzian function. Here we show the distribution of the full width at half maximum (FWHM) of the fitted functions (**a**). We also show each selected cluster alongside the fitted function and

the corresponding FWHM (**b**). The clusters in **b** represent raw (unsmoothed) image data and are plotted with a color scale normalized to the minimal and maximal pixel value within each panel. Scale bar = 100 nm. While we do not know the size distribution of transferrin receptor clusters, we anticipate that many clusters are smaller than the resolution limit of our microscope. We therefore use the size-estimates from the smallest clusters as an estimate of our resolution. A significant fraction of the size distribution is below 50 nm, suggesting that our live-cell image resolution is below 50 nm. See the **Supplementary Note 1** detailing our choice of pixel size and other imaging parameters.

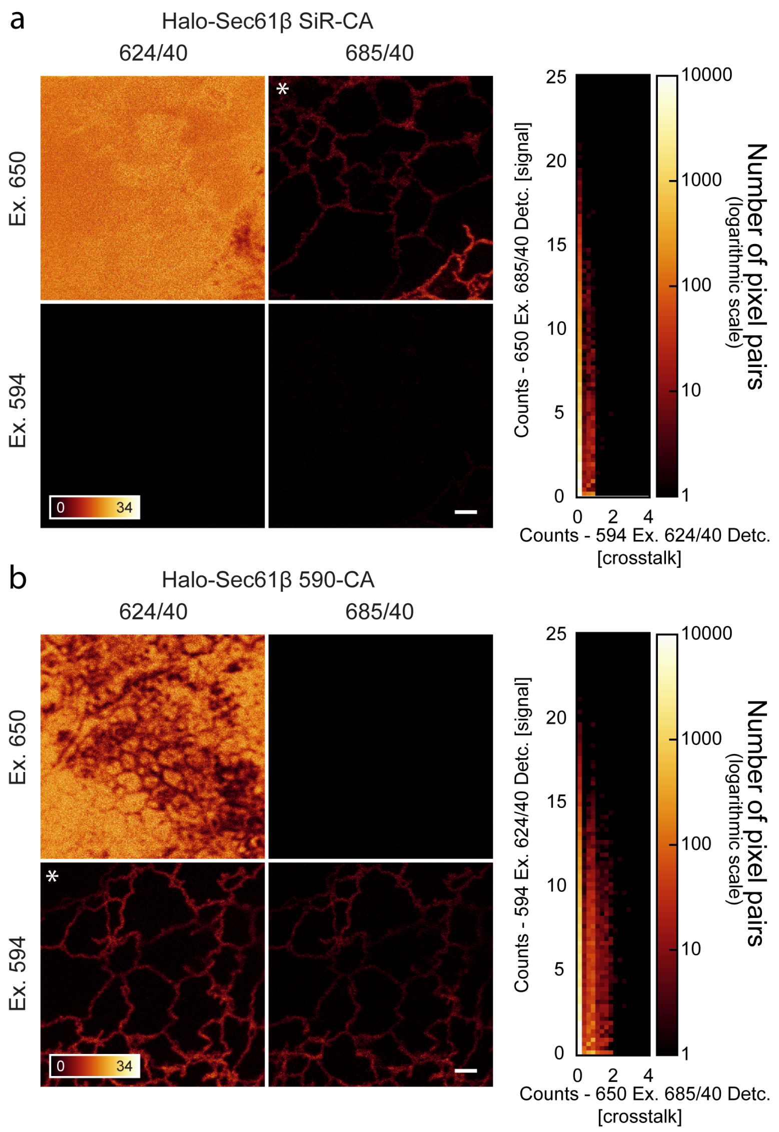

Supplementary Figure 4: Absence of crosstalk between the two channels. COS-7 cells expressing Halo-Sec61β were labeled with either (**a**) SiR-CA or (**b**) 590-CA. Cells were then fixed with 3% PFA and 0.1% glutaraldehyde. STED images of both samples were acquired using both excitation sources (594 nm and 650 nm) and detection windows (624/40 nm and 685/40 nm). When imaging with the 650 nm excitation laser and detecting in the 685/40 nm range only signal from the SiR dye is detected. Likewise, when imaging with the 594 nm excitation laser and detecting in the 624/40 nm range only signal from the ATTO590 dye is detected. The two crosstalk-free detection channels are marked by asterisks (*). Scale bars = 1 μm. The histograms of pairs of pixel values in the two detection channels on the right allow to quantify the crosstalk between the two detection channels. Linear regression of the data in the histograms (signal vs. crosstalk) yielded crosstalk estimates of 0.4% for SiR (signal in the 624/40 nm channel relative to the 685/40 nm) and 1.0% for ATTO590 (signal in the 685/40 nm channel relative to the 624/40 nm channel).

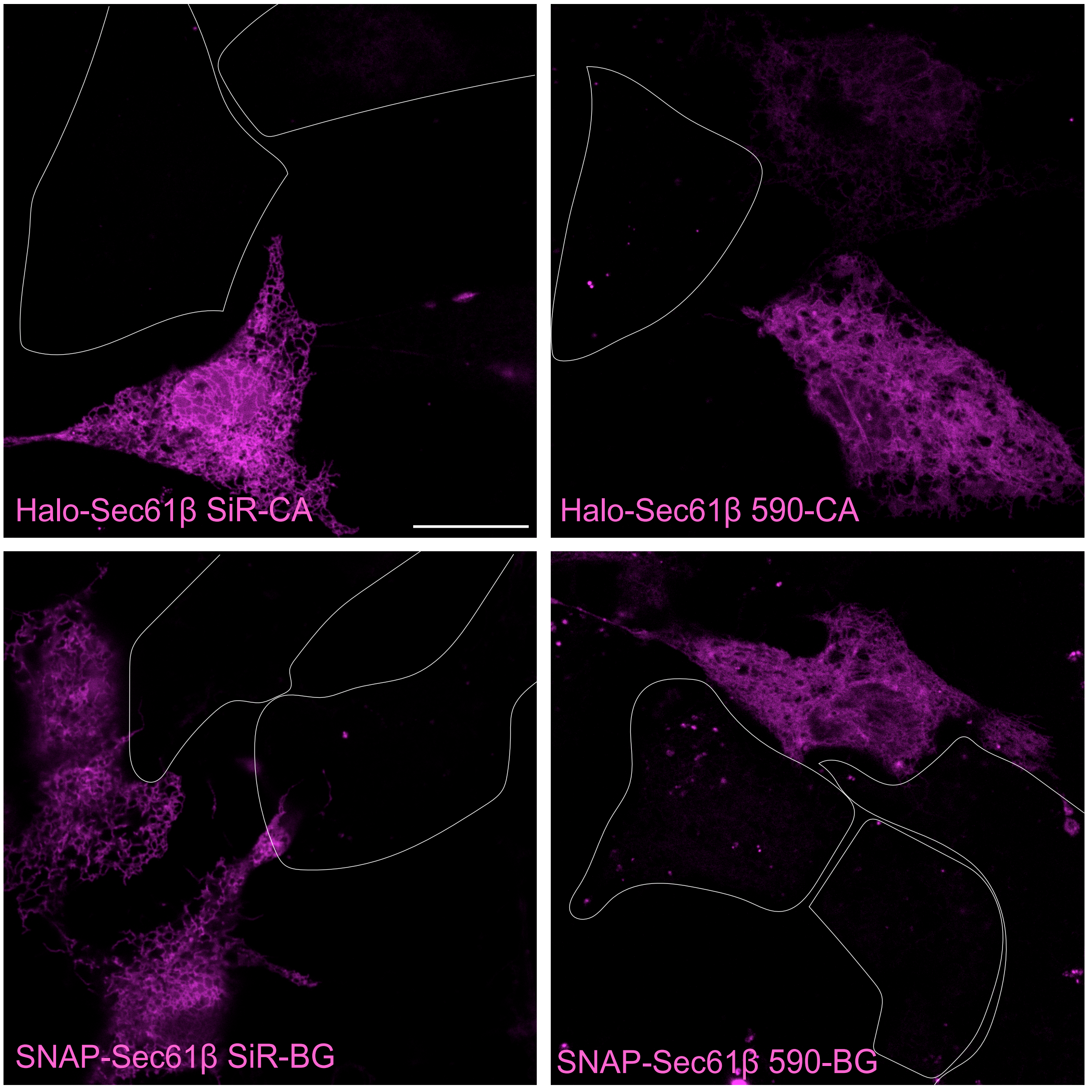

Supplementary Figure 5: Negligible background. Halo and SNAP substrates of both SiR and ATTO590 show no or little background and no unspecific labeling of intracellular membranes. We transfected COS-7 cells with Halo-Sec61β and SNAP-Sec61β and labeled them with Halo and SNAP substrates of both SiR and ATTO590. Wider fields of view which show non-transfected and transfected cells were recorded on a Leica SP5 confocal microscope. The non-transfected cells show no or very little background due to endocytosis of the dyes. Scale bar = 20 µm.

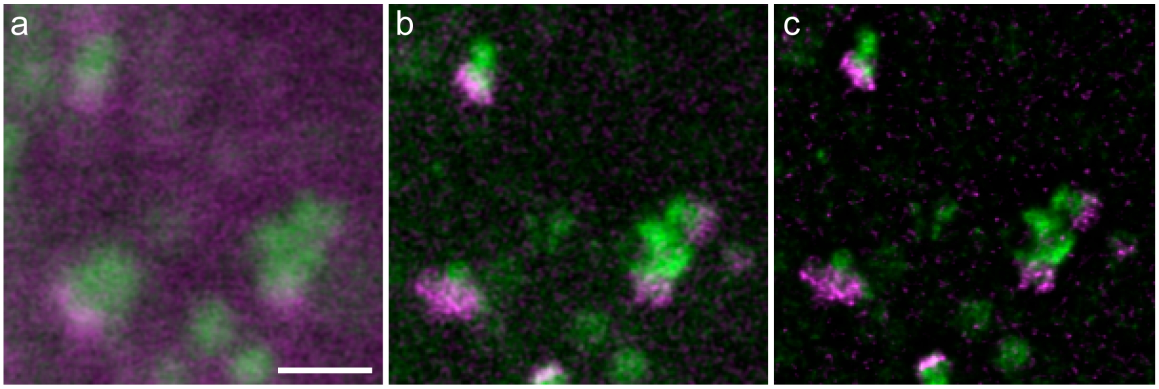

Supplementary Figure 6: Confocal vs. STED imaging of Golgi ministacks. Live nocodazole-treated HeLa cells expressing ManII-Halo labeled with SiR-CA (magenta) and SNAP-Rab6 labelled with 590-BG (green). (**a**) Confocal, (**b**) STED, (**c**) STED deconvolved as described in **Supplementary Methods**. Scale bar = 1 µm.

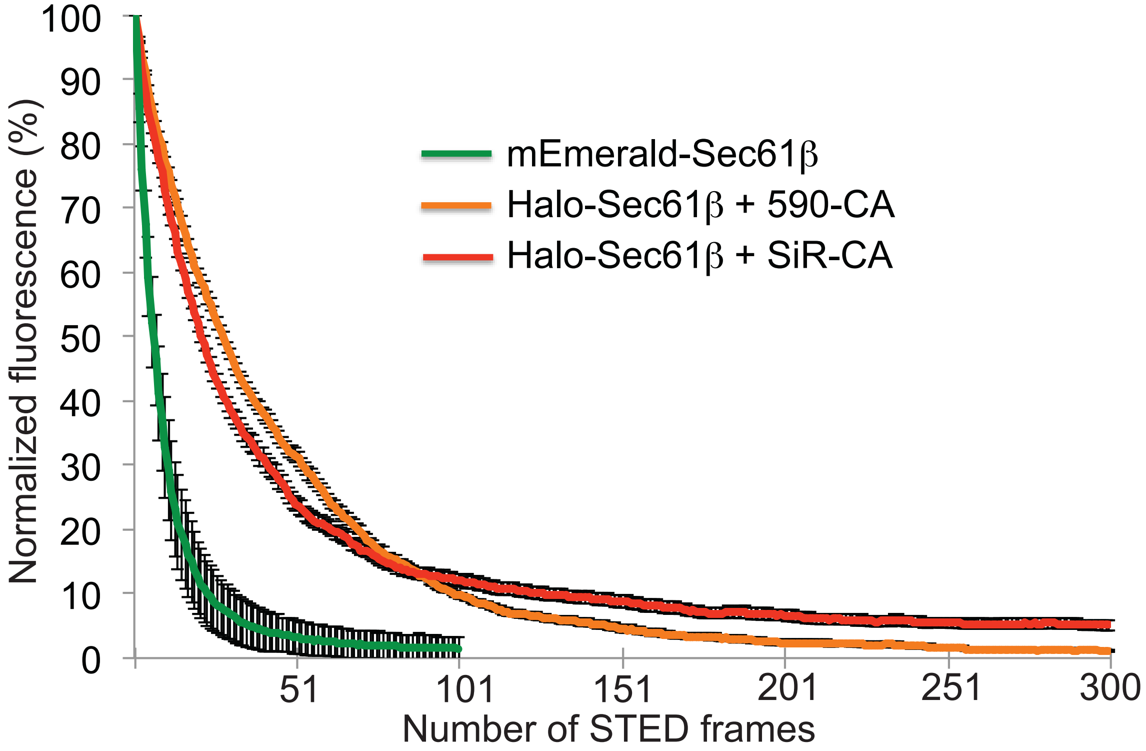

Supplementary Figure 7: Bleaching experiments show less photobleaching of ATTO590 and SiR compared to the fluorescent protein mEmerald. Using a commercial STED nanoscope (SP8, Leica) we imaged living COS-7 cells expressing either mEmerald-Sec61β or Halo-Sec61β labeled with SiR-CA and 590-CA. For a reasonably fair comparison, we chose wavelengths and laser powers to optimize image quality in STED imaging (**Supplementary Methods**). The following wavelengths were used: mEmerald: exc.: 485 nm, STED: 592 nm, det.: 505-550 nm; ATTO590: exc.: 594 nm, STED: 775 nm, det.: 604-644 nm; SiR: exc.: 650 nm, STED: 775 nm, det.: 665-705 nm. While this comparison does not represent an exhaustive study, it indicates that the fluorescent protein mEmerald bleaches significantly faster than the two fluorescent dyes in STED nanoscopy. Error bars represent the standard error across data obtained from 10 cells per trace.

Supplementary Note 1

While we can obtain 20-30 nm resolution with our STED nanoscope (**Supplementary Figure 2**), we chose a pixel size of 20 nm for live-cell STED imaging, thereby allowing a resolution no better than 40 nm according to the Nyquist limit. This decision was based on a compromise between imaging speed and photobleaching on one hand and spatial resolution on the other hand. Larger pixel sizes allow to image the same field of view significantly faster, because fewer pixels have to be recorded. This, plus the fact that the STED laser power can be reduced (since effective focus sizes below the Nyquist resolution limit are of no advantage), decreases the laser light exposure and, thereby, reduces bleaching and allows to acquire longer image sequences.

When considering temporal resolution in a STED nanoscope, it is important to note an inherent advantage of a point scanning geometry over widefield recording, namely that small, local structures are imaged much quicker than the time it takes to record a full frame. While it takes about 2 s to scan a ~10 x 10 µm field of view (**Fig. 1-4**), structures on the 250 nm scale in the same image are fully scanned in about 50 ms. Each individual line of the image (which is accumulated from 32 line scans per color channel) is finished scanning in ~4 ms. For this reason, small fast-moving objects are not nearly as susceptible to motion blur as they would be in a widefield microscope. Hence, when observing local dynamic events limited to small sections of an image, such as the image sequences shown in the subpanels of **Figure 1-4**, the data should be interpreted as short, sub-second snapshots interleaved by the 2 s it takes to scan the full field of view. While the point scanning geometry inherently suppresses motion blur, it is, however, important to be aware that the top and bottom of an image are out of sync by nearly 2 s. This can lead to motion artifacts when imaging large structures which extend along the slow scanning image axis. These characteristics can lead to artifacts as shown, for example, in **Supplementary Movie 1**. Here, the fast moving ER tubules appear jagged only along the slow scanning axis of the image.

Supplementary Note 2

Recombinant plasmids

To generate Halo-tagged Sec61β, mEmerald-Sec61β-C-18 (Addgene plasmid #54249, Davidson lab, unpublished) was cut with NheI and BglII and Halo tag was inserted as a NheI-BglII fragment. Halo was amplified with the following oligos:

Halo-s 5’-cctaggctagcatggcagaaatcggtactggctttccat-3’ and
Halo-as 5’- gagccagatcttgatccggagccggaaatctcgagcgtcgacagccagcg-3’.

To generate SNAP-tagged OMP25, the prHom-1 vector (Clontech) vector was linearized with XbaI and BamHI. SNAP and OMP25 were then inserted as XbaI-SpeI and SpeI-BamHI fragments, respectively. SNAP was amplified from pSNAPf (New England Biolabs) with the following oligos:

SNAP-s 5'-atcatctctagagaacaaaaacttatttctgaagaagatctgatggacaaagactgcgaa-3' and
SNAP-as 5'-ggcaagcctgggctgggtactagtcatcat-3'.

OMP25 was amplified from FRB-GFP-OMP25 with the following oligos:

OMP25-s 5’-atcatcactagtcaggtgcagaatggacctatagga-3' and
OMP25-as 5'-ccttgctgttcacagtctccaattggatcccatcat-3'.

SNAP-Rab6 was generated by replacing GFP with SNAP as a NheI-XhoI fragment in GFP-Rab6. SNAP was amplified with

SNAP-s 5’-ggatcgtcgctagcgatatcggcgcgccagcatttaaat-3’ and
SNAP-as 5’-cgaagcttgagctcgagatctgagtccggaacccagcccaggcttgcccagtctgtgg-3’.

GFP-Rab6 was initially generated by digesting pEGFP-C1 with EcoRI and BamHI and inserting an EcoRI-BamHI Rab6 fragment generated with the following oligos:

Rab6-s 5’-gcgtcgaattctatgtccacggcggagacttcggg-3’ and
Rab6-as 5’-tcgaacggatccttagcaggaacagcctccttcactgac-3’.

To generate ManII-Halo, prHom-1 vector (Clontech) was linearized with EcoRI and SpeI, ManII and Halo were then inserted as EcoRI-XbaI and XbaI-SpeI fragments respectively. ManII was amplified from ManII-GFP[1] with the following oligos:

ManII-s 5'-atcatcgaattcatgaagttaagtcgccagttc-3' and
ManII-as 5'- gtctgtggaggacggcccgcggtctagacatcat-3'.

Halo was amplified using the following oligos:

Halo-s 5'-atcatctctagaatggcagaaatcggtactggcttt-3' and
Halo-as 5'- gctgtcgacgctcgagatttccggagtactcatcat-3'.

To generate TfR-FM4-Halo, the pC4S1-FM4 vector[2] was linearized with SpeI and BamHI. The Halo fragment was then cloned with a GGSG linker (GGCGGAAGCGGC) into the pC4S1-hTFRc-FM4 vector by in-fusion cloning (Takara Bio Inc.). Halo was amplified from Halo-tag vector (pfc16K, Promega) using the following oligos:

Halo-s 5’-aaactggaaactagtggcggaagcggcgaggatctgtactttcagagcg-3’ and
Halo-as 5’-aagttctcaggatccttaaccggaaatctccagagtagac-3’.

To generate SNAP-CLC, mEos3.2-CLC[3] was linearized with AgeI and XhoI and SNAP was inserted as an AgeI/XhoI fragment. SNAP was amplified with the following oligos:

SNAP-s 5'-atcatctctagaaccggtatggacaaagactgcgaa-3' and
SNAP-as 5'-ggcaagcctgggctgggtctcgagcatcat-3'.

To generate signal sequence-SNAP-KDEL, the prHom-Sec1 vector (Clontech) was linearized with XbaI and BamHI. SNAP-KDEL was amplified as a XbaI/BamHI fragment with the following oligos:

SNAP-s 5’-ggcagtgcctctagagacaaagactgcgaaatgaagcgcac-3’ and
SNAP-KDEL-as 5’-tcgcatggatccctacaattcgtccttacccagcccaggcttgcccagtctgtgg-3’.

Confocal microscopy
Confocal images in **Supplementary Figure 5** were acquired with a commercial Leica TCS SP5 microscope. 595 nm and 633 nm wavelength were used to excite ATTO590 and SiR, respectively. Fluorescence was detected using avalanche photodiodes. Imaging was performed with a 100X/1.4 NA oil immersion objective lens.

STED imaging on Leica TCS SP8 STED 3X
STED imaging in **Supplementary Figure 7** was performed with a Leica TCS SP8 STED 3X. mEmerald, ATTO590 and SiR were excited with 485 nm, 594 nm and 650 nm wavelength respectively. A 592-nm laser was used for depletion of mEmerald while a 775-nm laser was used for depletion of ATTO590 and SiR. HyD detectors were used for the detection of all three channels. The detection windows were adjusted to 505-550 nm, 604-644 nm and 665-705 nm for mEmerald, ATTO590 and SiR, respectively.

Image processing and deconvolution
To reduce noise, STED movie sequences were deconvolved using the Richardson-Lucy algorithm[4, 5] implemented in python and available as part of the python-microscopy package (code.google.com/p/python-microscopy). Specifically, each 2D frame was deconvolved using a 2D Lorentzian approximation to the STED imaging point spread function (PSF)[6-8] with a full width at half maximum (FWHM) estimated from STED images of sub-diffraction sized fluorescent beads. Our principal goal of noise reduction was facilitated by initializing the algorithm with a uniform prior and terminating after a relatively low number of 10 iterations, both of which have a strong regularizing effect. Under these conditions we see a significant improvement in the signal-to-noise ratio, but little to no change in resolution. When used like this the algorithm is also relatively insensitive to the small variations in the FWHM of the PSF that may result from differing imaging conditions.

Raw images were corrected for bleaching using the exponential fit method in ImageJ[9] prior to deconvolution. Images and raw movies were smoothed with a Gaussian filter with 1 pixel standard deviation using ImageJ.

Materials, reagents, and instrumentation for chemical synthesis
ATTO590-NHS was obtained from Sigma-Aldrich as mixture of 5- and 6-isomers (79636, Sigma). UPLC and HR-LCMS was performed on a Waters Xevo Q-TOF LCMS with Electrospray Ionization using a Waters Acquity UPLC BEH300 C18 1.7 µm (186003686) column (solvent A: H_2O with 0.1% formic acid; solvent B: MeCN with 0.1% formic acid). Semi-preparative RP-HPLC was carried out on an Agilent 1260 Infinity instrument using a Vydac 219TP Diphenyl Column (250 mm x 10 mm) column from Grace (solvent A: H_2O with 1% MeCN and 0.1% TFA; solvent B: MeCN with 0.1% TFA).

Supplementary Note 3

Synthesis of ATTO590-CA (3)

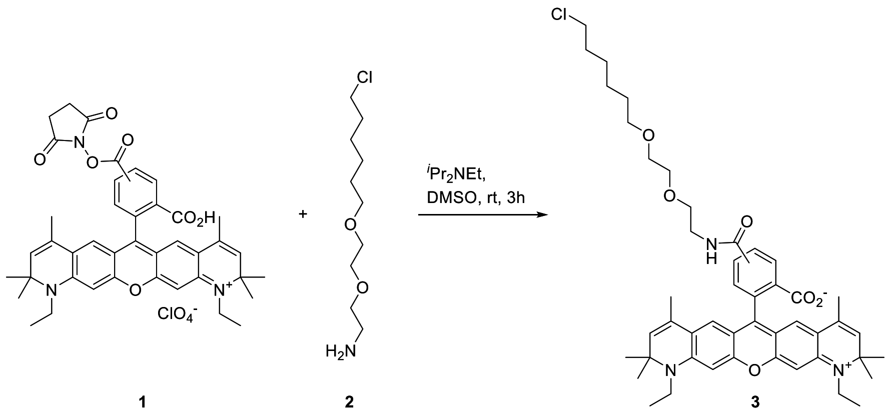

1 **2** **3**

ATTO590-NHS (**1**) (1.0 mg, 1.3 µmol, 1.0 eq) was dissolved in DMSO, ^{i}Pr$_2$NEt (2.2 µL, 13 µmol, 10 eq) and chloroalkane **2** (synthesized following a previously published procedure[10]) (0.99 mg, 4.4 µmol, 3.5 eq) were added. The reaction mixture was stirred for 3 h under the exclusion of light. The reaction mixture was directly subjected to RP-HPLC purification. The title compound **3** was obtained as a solid after evaporation under reduced pressure of the collected HPLC fractions.
HRMS (ESI): *m/z* calc. for $C_{47}H_{59}ClN_3O_6^+$: 796.4087; found: 796.4073 (Δ = -1.8 ppm).
UPLC: t_R=1.92 min; gradient: 0 min: 5% B, 1 min: 5% B, 1.6 min: 95% B, 3.0 min: 95% B; C18 column) .

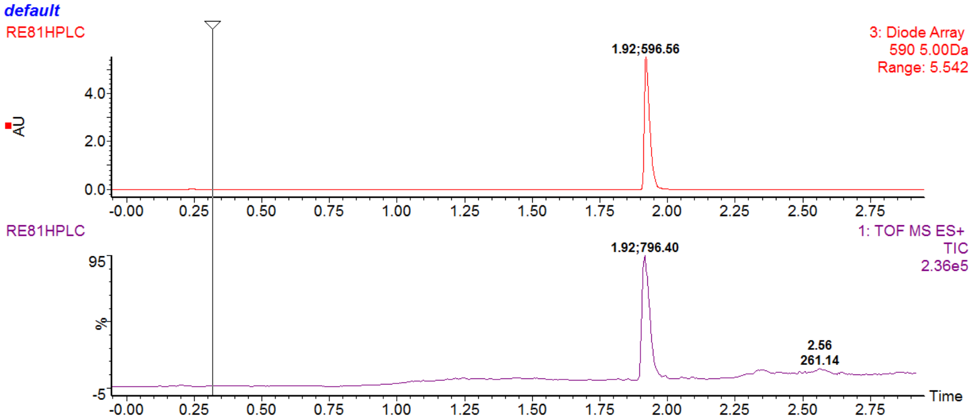

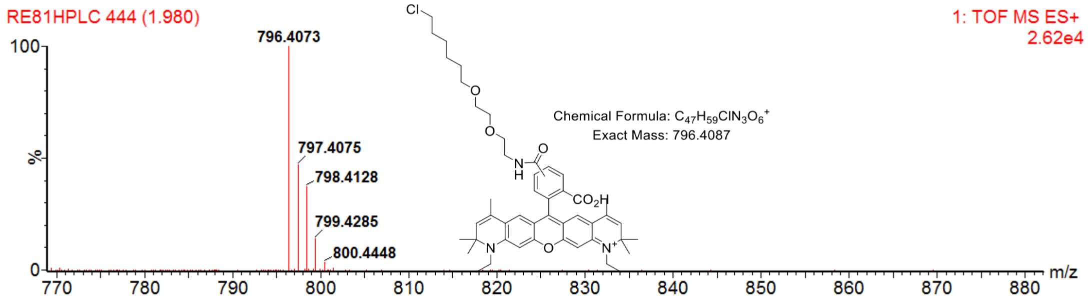

Synthesis of ATTO590-BG (5)

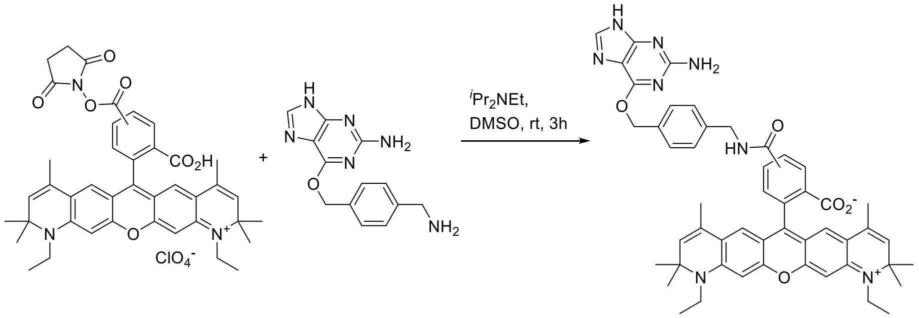

1 4 5

ATTO590-NHS (**1**) (1.0 mg, 1.3 µmol, 1.0 eq) was dissolved in DMSO, $^{i}Pr_2NEt$ (2.2 µL, 13 µmol, 10 eq) and benzoguanidine **4** (synthesized following a previously published procedure[11]) (1.4 mg, 5.1 µmol, 4 eq) were added. The reaction mixture was stirred for 3 h under the exclusion of light. The reaction mixture was directly subjected to RP-HPLC purification. The title compound **5** was obtained as a solid after evaporation under reduced pressure of the collected HPLC fractions.

HRMS (ESI): *m/z* calc. for $C_{50}H_{52}N_8O_5^{2+}$: 422.2025; found: 422.2010 (Δ = -3.6 ppm).

UPLC: t_R=1.59 min; gradient: 0 min: 5% B, 1 min: 5% B, 1.6 min: 95% B, 3.0 min: 95% B; C18 column).

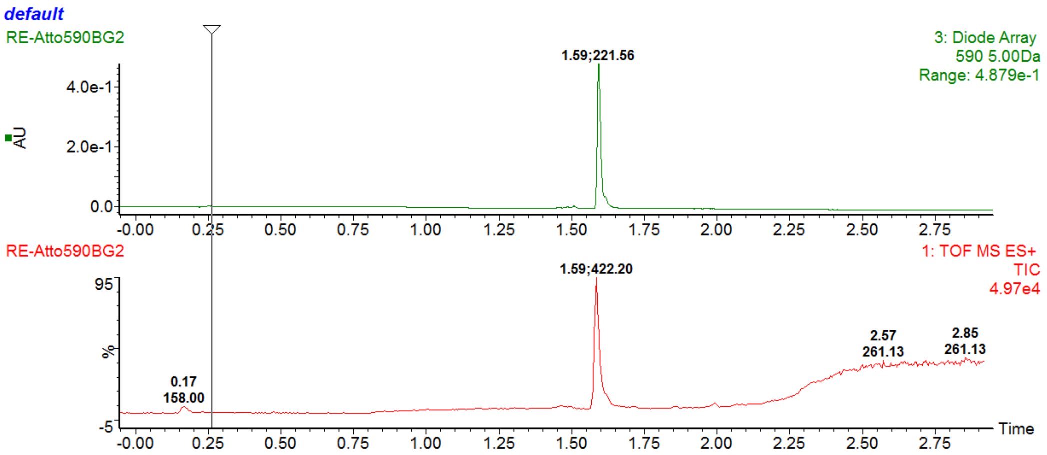

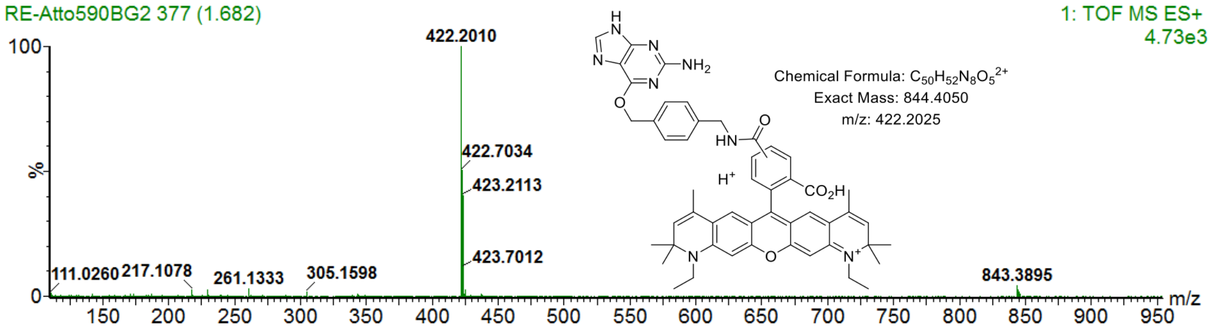

Supplementary Reference

1. Lavieu, G., Zheng, H. & Rothman, J.E. Stapled Golgi cisternae remain in place as cargo passes through the stack. *Elife* **2** (2013).
2. Rivera-Molina, F. & Toomre, D. Live-cell imaging of exocyst links its spatiotemporal dynamics to various stages of vesicle fusion. *Journal of Cell Biology* **201**, 673-680 (2013).
3. Huang, F. *et al.* Video-rate nanoscopy using sCMOS camera-specific single-molecule localization algorithms. *Nature Methods* **10**, 653-+ (2013).
4. Lucy, L.B. An iterative technique for the rectification of observed distributions. *Astronomical Journal* **79**, 745-754 (1974).
5. Richardson, W.H. Bayesian-Based Iterative Method of Image Restoration. *J Opt Soc Am* **62**, 55-& (1972).
6. Lukinavicius, G. *et al.* Fluorogenic probes for live-cell imaging of the cytoskeleton. *Nature methods* **11**, 731-733 (2014).
7. Matkovic, T. *et al.* The Bruchpilot cytomatrix determines the size of the readily releasable pool of synaptic vesicles. *The Journal of cell biology* **202**, 667-683 (2013).
8. Lukinavicius, G. *et al.* A near-infrared fluorophore for live-cell super-resolution microscopy of cellular proteins. *Nat Chem* **5**, 132-139 (2013).
9. Abramoff, M.D., Magalhaes, P.J., Ram, S.J. Image Processing with ImageJ. *Biophotonics International* **11**, 36-42 (2004).
10. Los, G.V. *et al.* HaloTag: A novel protein labeling technology for cell imaging and protein analysis. *ACS Chem. Biol.* **3**, 373-382 (2008).
11. Keppler, A. *et al.* A general method for the covalent labeling of fusion proteins with small molecules in vivo. *Nature biotechnology* **21**, 86-89 (2003).
